# Association between Coronary Artery Spasm and the risk of incident Diabetes: A Nationwide population-based Cohort Study

**DOI:** 10.7150/ijms.57987

**Published:** 2021-05-03

**Authors:** Ming-Jui Hung, Nen-Chung Chang, Patrick Hu, Tien-Hsing Chen, Chun-Tai Mao, Chi-Tai Yeh, Ming-Yow Hung

**Affiliations:** 1 Division of Cardiology, Department of Medicine and Community Medicine Research Center, Chang Gung Memorial Hospital, Keelung, Chang Gung University College of Medicine, Keelung City, Taiwan.; 2 Division of Cardiology, Department of Internal Medicine, Taipei Medical University Hospital, Taipei, Taiwan.; 3 Division of Cardiology, Department of Internal Medicine, School of Medicine, College of Medicine, Taipei Medical University, Taipei, Taiwan.; 4 Taipei Heart Institute, Taipei Medical University, Taipei, Taiwan.; 5 University of California, Riverside, Riverside, California, USA.; 6 Department of Cardiology, Riverside Medical Clinic, Riverside, California, USA.; 7 Department of Medical Research and Education, Shuang Ho Hospital, Taipei Medical University, New Taipei City, Taiwan.; 8 Department of Medical Laboratory Science and Biotechnology, Yuanpei University of Medical Technology, Hsinchu City 300, Taiwan.; 9 Division of Cardiology, Department of Internal Medicine, Shuang Ho Hospital, Taipei Medical University, New Taipei City, Taiwan.

**Keywords:** coronary artery spasm, insulin resistance, risk factors, sex difference, type 2 diabetes

## Abstract

**Background:** Non-diabetic coronary artery spasm (CAS) without obstructive coronary artery disease increases insulin resistance. We investigated the risk of incident type 2 diabetes (diabetes) associated with CAS.

**Methods:** Patient records were retrospectively collected from the Taiwan National Health Insurance Research Database during the period 2000-2012. The matched cohorts consisted of 12,413 patients with CAS and 94,721 patients in the control group.

**Results:** During the entire follow-up, the incidence of newly-diagnosed diabetes was 22.2 events per 1000 person-years in the CAS group and 13.9 events per 1000 person-years in the control group. The increased risk of CAS-related incident diabetes was observed regardless of sex and length of follow-up. The median time to incident diabetes was 2.9 and 3.5 years in the CAS and the control group (*P* <0.001), respectively, regardless of sex. Although age did not affect the risk of CAS-related incident diabetes, the risk was less apparent in the subgroups of male, dyslipidemia, chronic obstructive pulmonary disease, stroke, gout and medicated hypertension. However, CAS patients aged <50 years compared with patients ≥50 years had a greater risk of incident diabetes in females but not in males. Older CAS patients developed diabetes in a shorter length of time than younger patients.

**Conclusion:** CAS is a risk factor for incident diabetes regardless of sex. However, females aged <50 years have a more apparent risk for CAS-related diabetes than old females, which is not observed in males. The median time of 2.9 years to incident diabetes warrants close follow-up.

## Introduction

Coronary artery spasm (CAS), an inflammatory nonobstructive coronary disease characterized by elevated C-reactive protein (CRP) levels, plays an important role in myocardial ischemia, infarction, and sudden death 1,2. The 2 important risk factors for CAS 1. smoking and CRP, are strongly correlated with insulin resistance 3. Among non-diabetic patients, a significant positive association has been demonstrated between CAS and insulin resistance 4, leading to blood flow-dependent decrease in glucose disposal and hyperglycemia 5. Although inflammation contributes to synergistic coupling of endothelial dysfunction and insulin resistance 5, treatment with insulin sensitizers to decrease vascular disease has yielded mixed results, perhaps because insulin resistance may promote not only obstructive atherosclerosis 6, but also CAS. On the other hand, while racial heterogeneity exists in CAS 1 and insulin resistance 7, the prevalence of type 2 diabetes (diabetes) is not substantially different between Japanese 8 and Whites 9. Because diabetes can occur in the absence of insulin resistance, insulin resistance per se is an insufficient cause of diabetes 10. In light of such a scenario, CAS is potentially important in modulating the development of diabetes in Asians. It is well established that a long prodromal stage of ≥10 years 11 exists before incident diabetes, suggesting that CAS may start long before the onset of diabetes.

The relationship between cardiovascular disease and glycemia is progressive and begins at a fasting glucose level of 70 mg/dL and a hemoglobin A1C level of 5%, values well below those seen in diabetes 12. Furthermore, the strong association of insulin and endothelial signaling disturbances contributes to the mechanisms of homeostasis between endothelial vasodilation-vasoconstriction 13. While insulin resistance and elevated fasting glucose appear decades before incident diabetes 6,11, the effects of CAS on the risk of incident diabetes in patients without obstructive coronary artery disease have not been investigated. Whether the impact of CAS on incident diabetes is unique, or just a general effect of stress is unknown. Among healthy non-diabetic people, insulin resistance is lower in women than in men 14, reflecting estrogen-facilitated glucose homeostasis in women compared to men. In addition, sex-related differences are found in the incidence of diabetes 15. We, therefore, sought to determine the risk of prior CAS without obstructive coronary artery disease, with and without regard to sex, on incident diabetes in a nationwide population-based cohort and retrospective case-control study.

## Material and methods

### Study Design and Subjects

This was designed as a prospective cohort study of retrospectively-collected data from the National Health Insurance Research Database (NHIRD), which was launched by Taiwan's National Health Institute (NHI) Program in March 1995 and provided approximately 99.8% coverage for the 23.9 million citizens in Taiwan. The NHIRD contains data for outpatient and inpatient services, including diagnoses, medications, interventions, operations, hospitalizations and emergency visits 16,17. Diagnosis is registered using the International Classification of Diseases, Ninth Revision, Clinical Modification (ICD-9-CM) codes 16,17. The definition of a chronic disease by using 1 inpatient or 3 outpatient diagnoses is a conventional and common usage in Taiwan NHIRD studies. This definition of CAS has been reported in our previous work 18. The present study was approved by the Institutional Review Board of Chang Gung Memorial Hospital (ID: 103-0248B) with no informed consent required because of the secondary nature of the deidentified data in the retrospective study design.

The data of the laboratory variables of the NHIRD are not stored in digital format. Hence, in order to provide laboratory values about incident diabetes risk, we conducted a single Shuang Ho hospital study from September 2008 to April 2015 in 252 non-diabetic patients, who had suspected ischemic heart disease, no angiographic evidence of obstructive coronary artery disease, and were subjected to intracoronary methylergonovine testing (Supplementary Table S1). The diagnostic criteria of CAS were angina at rest associated with electrocardiographic ST-segment elevation or depression, which was relieved by sublingual nitroglycerin, no obstructive coronary artery disease on coronary angiography after intracoronary nitroglycerin administration, and a positive intracoronary methylergonovine provocation testing result 19, which was not necessary for patients with ST-segment elevation during episodes of angina and no obstructive coronary artery disease 2. At baseline, we collected data on demographic information, coexisting illnesses, anthropometric values, use of medications, and laboratory values. Current smoking was defined as having smoked a cigarette within 3 weeks of the cardiac catheterization. Patients underwent echocardiography, when heart rates were recorded, before coronary angiography and within 2 weeks of the last angina. The blood pressure levels were measured at the time of coronary angiography. Among these patients, 140 patients had CAS and 112 did not have CAS. This hospital study was approved by the Taipei Medical University-Joint Institutional Review Board (No. 201011004 version 1.3). All patients gave written informed consent.

**Figure 1:**
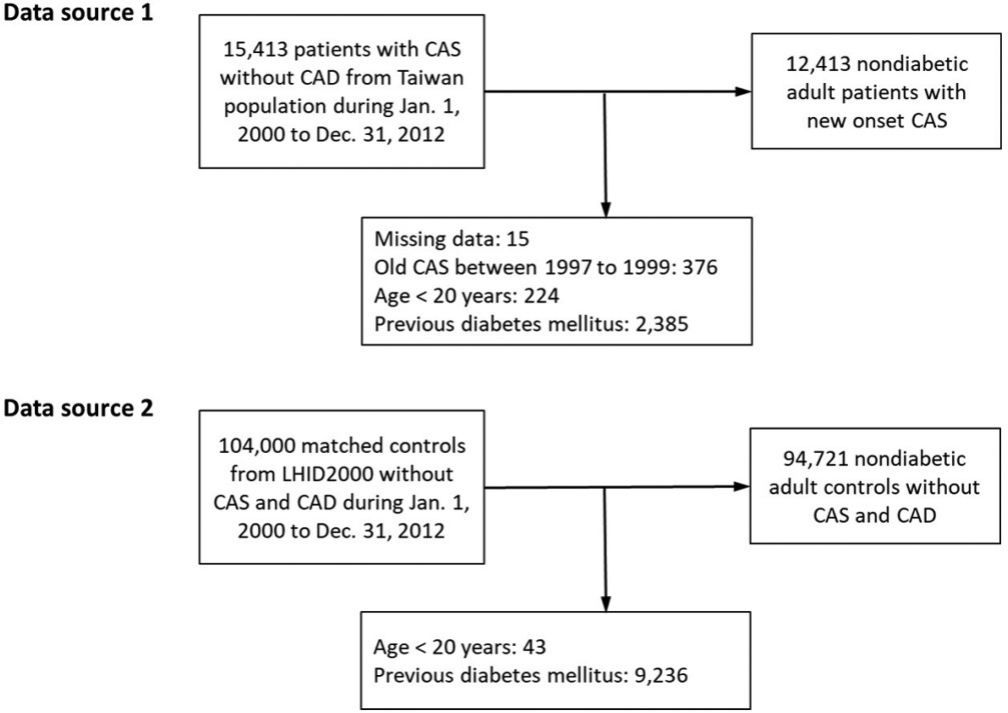
Study flow diagram. CAD: coronary artery disease; CAS: coronary artery spasm; LHID200: Longitudinal Health Insurance Database 2000.

The CAS group comprised adult patients (≥20 years) with newly-diagnosed CAS (ICD-9-CM code 4131), with at least 3 outpatient diagnoses or 1 inpatient diagnosis between January 1, 2000 and December 31, 2012 in the NHIRD of the entire Taiwanese population because the prevalence of CAS was not high. Subjects in the control group were identified from a subset of the NHIRD, the Longitudinal Health Insurance Database 2000, which consists of claims data from 1 million randomly sampled people who were alive during the year 2000 and had been followed up from 1997 to 2012. The Longitudinal Health Insurance Database 2000 is representative of the general Taiwanese population in terms of age, sex, or health care costs and has been validated by the National Health Research Institute 20. The control subjects did not have any previous diagnoses of CAS and obstructive coronary artery disease and were frequency-matched with the CAS group for sex, age, urbanization level, monthly income, and year of CAS diagnosis. The dates of diagnosis of new-onset CAS were assigned as index dates. Notably, the index date of the subjects in the control group was assigned from their matched counterparts in the CAS group. Subjects who had a diagnosis of diabetes before the index date in both the CAS and control groups were excluded (Figure 1).

### Provocative Protocol

The vasoreactivity was examined by the constrictor response to intracoronary methylergonovine during coronary angiography in the single center study. Coronary angiography was performed using the standard Judkins technique. Nitrates and calcium antagonists were withdrawn for ≥24 hours before the procedure. Left ventricular ejection fraction was calculated using Simpson's method. Obstructive coronary artery disease was defined as a ≥50% reduction in luminal diameter after administration of intracoronary nitroglycerin (100 μg) 19. If no obstructive coronary artery disease was found, intracoronary methylergonovine (Methergin®; Novartis, Basel, Switzerland) was administered stepwise (1, 5, 10, 30 μg) first into the right coronary artery and subsequently into the left coronary artery. CAS was defined as a >70% reduction in luminal diameter compared with postintracoronary nitroglycerin, with associated angina and/or ST depression or elevation 19. Provocation testing was stopped with an intracoronary injection of 50-200 μg of nitroglycerin (Millisrol®; G. Pohl-Boskamp, Hohenlockstedt, Germany).

### Covariates

The covariates were age at the index date, sex, urbanization level, monthly income, comorbidities (dyslipidemia, chronic obstructive pulmonary disease (COPD), stroke, gout, hepatitis C virus infection, depression, psychiatric disorders, medicated hypertension) and use of steroid. The comorbidities were verified by the ICD-9-CM diagnostic codes using at least 3 outpatient diagnoses or 1 inpatient diagnosis in the year before the index date. All the information about medication (anti-hypertensive drug or steroid) in the previous year before the index date were extracted from the claims data of outpatient visits or the refill for chronic illness in the pharmacy by using the Anatomical Therapeutic Chemical codes or the Taiwan NHI reimbursement code.

### Outcome

The incident diabetes was the outcome diagnosis, which was based on American Diabetes Association criteria and ICD-9-CM codes. The ICD-9-CM diagnostic code of diabetes has been validated in a previous Taiwan NHIRD study, by using ≥4 outpatient visits which corresponds to an accuracy of about 95.7% 21. The prescription of oral hypoglycemic agents corresponded to an accuracy of 99% 22. Therefore, the occurrence of diabetes required 4 outpatient diagnoses, with the use of any oral hypoglycemic agents. Mortality was defined by withdrawal from the NHI program 23. All patients were followed from the index date to the date of incident diabetes, date of withdrawal from the NHI program or December 31, 2013, whichever came first.

### Statistical Analyses

To minimize potential confounding when comparing the risk of incident diabetes between the CAS and control groups, we applied the inverse probability of treatment weighting (IPTW) based on the propensity score. The propensity score was estimated using a multivariable logistic regression model without considering the interaction effects in which the study group (1=CAS, 0=control) was regressed on all the covariates listed in Table 1, where the follow-up duration was replaced with the index date. We used the weight to estimate average treatment effect and adopted a stabilized weight to mitigate the impact of extreme estimated propensity scores. The balance of the covariates between the groups before and after IPTW was checked using the absolute value of standardized difference between the groups, where a value less than 0.1 was considered a negligible difference. In addition to IPTW, we also performed a propensity score matching with 1:1 ratio to evaluate the robustness of the results.

The risk of incident diabetes between the CAS and control groups was compared using Fine and Gray subdistribution hazard model of a competing risk, which considered all-cause mortality during follow-up. The index dates of the CAS group were based on the diagnosis dates, whereas those of the control group were assigned from the CAS group. Therefore, the control group could have fewer diagnoses at their index dates, which could have resulted in a detection bias and an overestimated effect. To rule out the potential impact of detection bias, patients who received a diagnosis of diabetes within 1 year after the index date were censored (the sensitivity analysis). The time to incident diabetes between the CAS and control groups was compared using the Mann-Whitney U-test. The trend of time to incident diabetes across age groups was tested using the linear contrast of general linear model.

Finally, subgroup analyses were done to evaluate the possible effect modification of the following pre-specified variables associated with incident diabetes based on a priori knowledge: age (<65 vs. ≥65 years), sex, the median age at natural menopause by 50 years in Taiwan 24, dyslipidemia, COPD, stroke, gout and medicated hypertension. A 2-sided *P* value <0.05 was considered significant and no adjustment of multiple testing (multiplicity) was made. All statistical analyses were performed using SAS version 9.4 (SAS Institute, Cary, NC), including the procedures of 'phreg' for the Fine and Gray model and the macro of '%cif' for generating cumulative incidence function under the Fine and Gray subdistribution hazard method.

For the single hospital study, continuous variables were expressed as mean ± standard deviation or median value and 25th, 75th percentiles, and log transformation was performed for variables with positive skewness for the subsequent Student's t tests between groups. Categorical variables were analyzed using the χ^2^ test. A 2-sided *P* value <0.05 was considered significant. All statistical analyses were performed with the statistical software package SPSS for Windows (Version 15.0, SPSS Inc., Chicago, IL).

## Results

### Patient Characteristics

A total of 12,413 nondiabetic CAS patients and 94,721 nondiabetic non-CAS control subjects were included in the final analysis (Figure 1). None of these patients has obstructive coronary artery disease. Before IPTW, the CAS group had lower urbanization levels and lower monthly income. The prevalence of dyslipidemia, COPD, gout, HCV infection, depression and medicated hypertension was higher in the CAS group than in the control group. In addition, the use of steroid was more common in the CAS group. After IPTW, none of the baseline characteristics were substantially different between groups with all of the absolute standardized difference values <0.1. The average follow-up was 6.2 years in both groups (Table 1). In the single hospital study, levels of fasting glucose and glycated hemoglobin were significantly higher in the CAS group (Supplementary Table S1).

### Sex-Specific Incidence of Diabetes Associated with CAS

**Figure 2:**
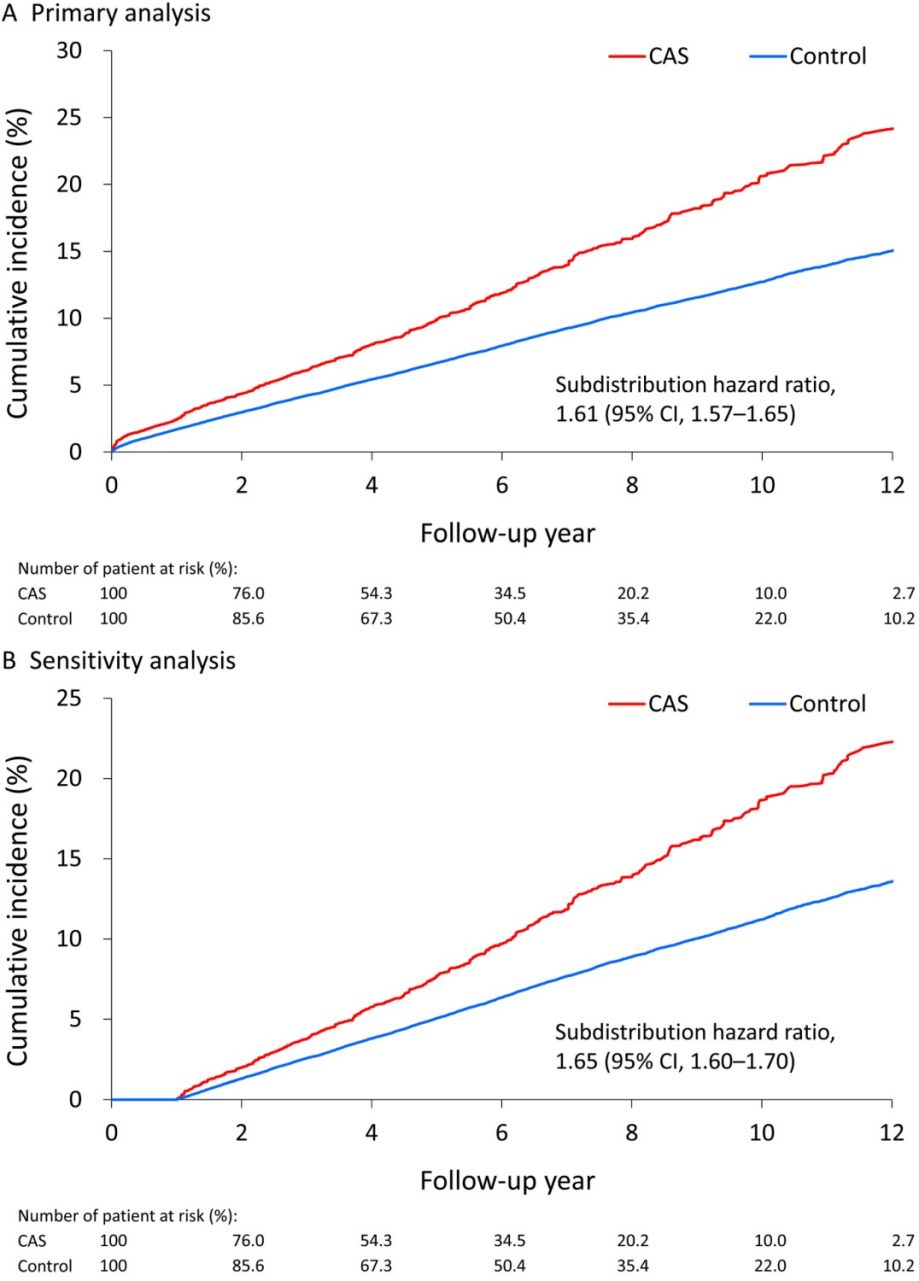
** Cumulative incidence of diabetes associated with CAS. (A)** The cumulative incidence of diabetes in the nondiabetic patients with CAS and control subjects in the primary analysis by inverse probability of treatment weighting. **(B)** The sensitivity analysis by censoring patients diagnosed as incident diabetes within 1 year after the index date. CAS: coronary artery spasm.

About 11.6%, 11.4% and 11.7% of all, male and female patients with CAS, respectively, developed incident diabetes during a mean follow-up of 6.2 years. During the entire follow-up, the incidence of diabetes was 22.2 events (95% confidence interval [CI], 21.8-22.6) per 1000 person-years in the CAS group and 13.9 events (95% CI, 13.6-14.2) per 1000 person-years in the control group. The annual incidence of diabetes was 2.16% in males with CAS and 2.26% in females with CAS. The increased risk of diabetes by CAS was observed at the first year follow-up (subdistribution hazard ratio [SHR], 1.45; 95% CI, 1.37-1.54), 3-year follow-up (SHR, 1.39; 95% CI, 1.35-1.43), 5-year follow-up (SHR, 1.53; 95% CI, 1.48-1.58) and until the end of follow-up (SHR, 1.61; 95% CI, 1.57-1.65). The same results were observed when stratifying the analysis by sex (Table 2). After censoring patients who developed incident diabetes within 1 year after the index date in the sensitivity analysis, CAS was still associated with a higher risk of incident diabetes at the 3-year follow-up, 5-year follow-up and until the end of follow-up (Supplementary Table S2). While the highest incidence of CAS-related diabetes after age 90 years may be due to sparse data phenomenon, the incidence of sex-specific CAS-related diabetes increased with age until 80 years, and was higher in the middle-aged groups (50-80 years) (Supplementary Table S3). In addition, the results by using propensity score matching with 1:1 ratio were consistent as the primary analysis by IPTW (Supplementary Table S4-S5). The IPTW-adjusted cumulative incidence function of incident diabetes in the CAS and control groups were depicted (Figure 2A, 2B).

### Subgroup Analysis

**Figure 3:**
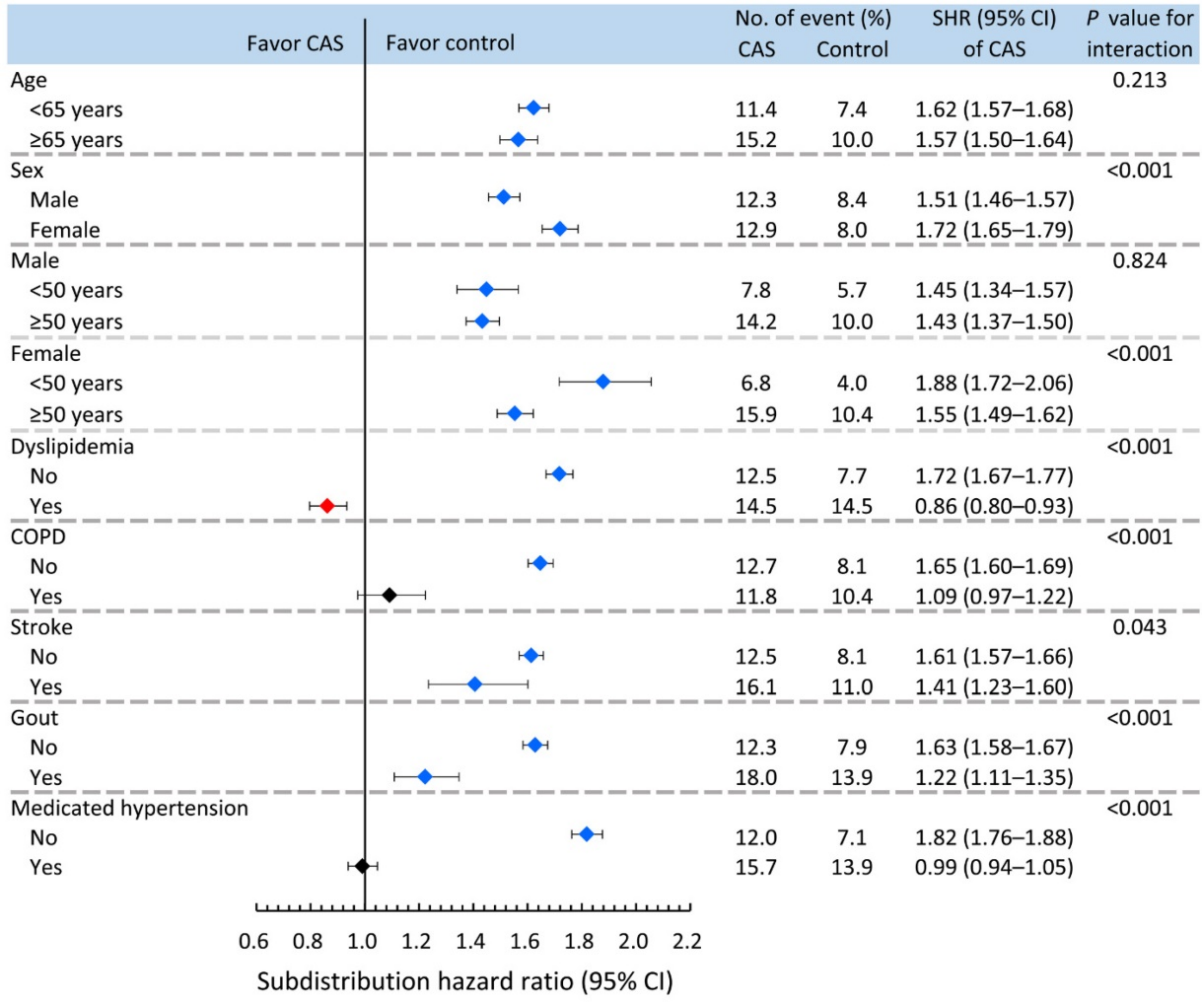
** Pre-specified nondiabetic subgroup analysis for comparing risk of incident diabetes between CAS and control subjects.** CAS: coronary artery spasm; COPD: chronic obstructive pulmonary disease.

Age, sex, dyslipidemia, COPD, stroke, gout and medicated hypertension were analyzed in the pre-specified subgroup analysis (Figure 3). The results showed that the increased risk of incident diabetes in association with CAS was less apparent in males and the dyslipidemia, COPD, stroke, gout and medicated hypertension groups (*P* for interaction <0.05). However, when stratified by age (dichotomized as <50 or ≥50 years) and sex, the observed increase in the risk of incident diabetes associated with CAS was greater in the younger than older females but not for males (*P* for interaction <0.001 and 0.824 in the female and male cohorts, respectively).

### Time to Incident Diabetes Stratified by Age and Sex

**Figure 4:**
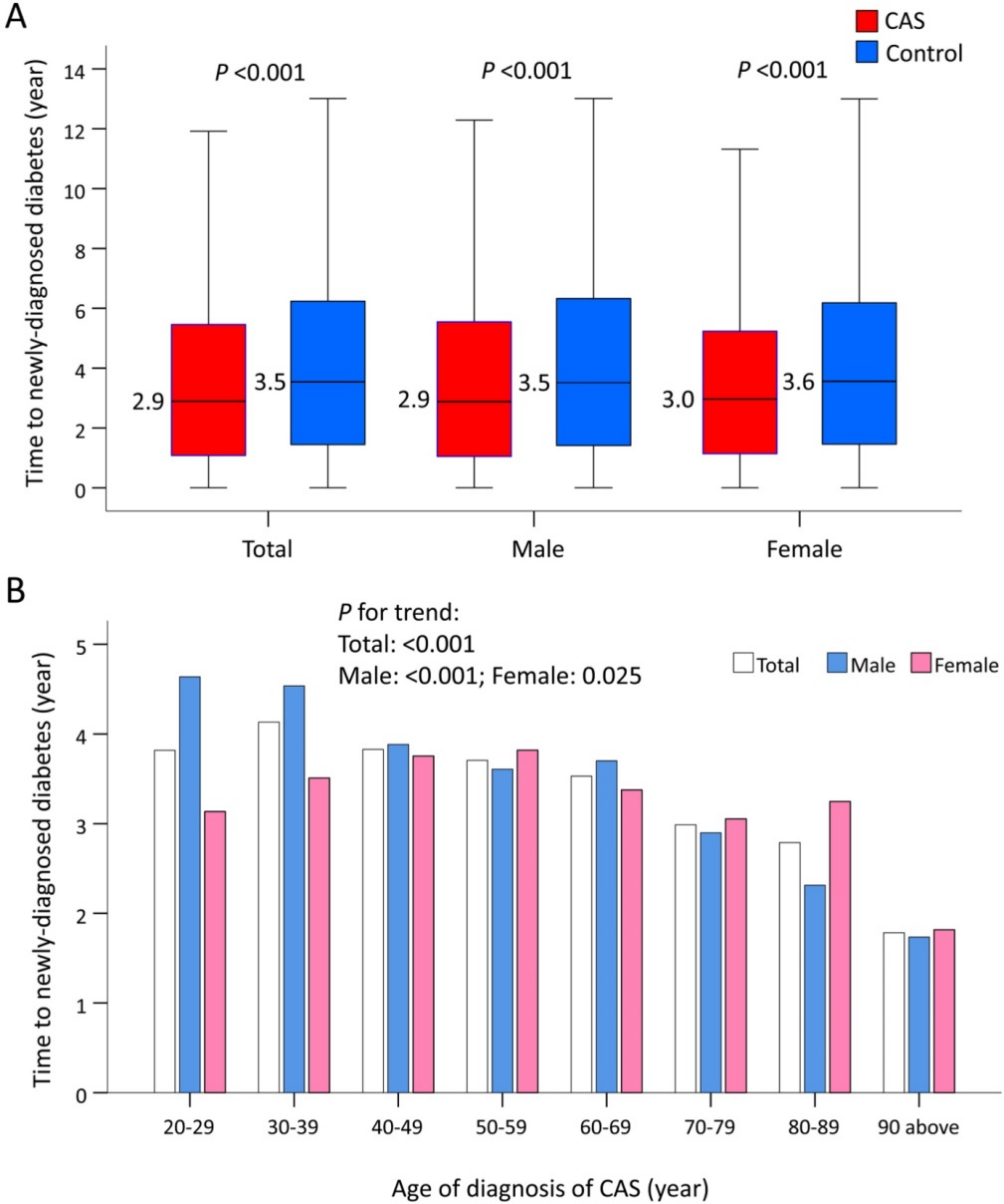
** Time to incident diabetes in patients with CAS and the control subjects before IPTW. (A)** Time to incident diabetes in patients with CAS and the control subjects by sex. **(B)** Time to incident diabetes in patients with CAS and the control subjects by sex and ages. CAS: coronary artery spasm; IPTW: inverse probability of treatment weighting.

The median time to newly-diagnosed diabetes was 2.9 years in the CAS group and 3.5 years in the control group (*P* <0.001), and did not vary by sex (Figure 4A). When stratified by age, older CAS patients compared with their younger counterparts had a much shorter length of time before a new case of diabetes was diagnosed (*P* trend <0.001; Figure 4(b)). When further stratified by age and sex, increasing age was associated with a shorter length of time before a diagnosis of diabetes in males with CAS (*P* trend <0.001). However, this phenomenon was less obvious in females with CAS (*P* trend = 0.025; Figure 4B).

## Discussion

In this large cohort study, we found that about 11.6% of the patients with CAS developed diabetes during a mean follow-up of 6.2 years. During the entire follow-up period, the respective annual incidence of diabetes was 2.22% in the CAS group and 1.39% in the control group. The median time to incident diabetes, which did not vary by sex, was 2.9 and 3.5 years in the CAS and the control groups, respectively. CAS was a risk factor for incident diabetes regardless of sex and the length of follow-up. Although age did not affect the risk of incident diabetes associated with CAS, the risk was less apparent in the subgroups of males and patients with dyslipidemia, COPD, stroke, gout, and medicated hypertension. CAS patients aged <50 years compared with patients ≥50 years had a greater risk of incident diabetes in females, but not in males. On the other hand, older male CAS patients compared with their younger counterparts developed diabetes in a shorter length of time. In contrast, this age phenomenon was not observed in the female CAS patients <50 years.

The incidence of newly-diagnosed diabetes is more than 3 times higher among patients with CAS than among general population in Taiwan 25 (22.2 per 1000 person-years vs. 6.2-7.4 per 1000 population). The sex-specific annual incidences of diabetes in CAS patients were 2.16% in males and 2.26% in females, which has not been reported previously. The annual risk of developing diabetes in people with normal glucose level is 0.7%, whereas patients with prediabetes have a yearly risk of up to 10% 26. Among non-diabetic stroke patients, the prevalence of fasting post-stroke hyperglycemia is 14.6% 27, and about 8% of survivors develop diabetes during a mean follow-up of 10 years 28, which is more than 2 times higher than expected compared with people from a Dutch general practitioner registry with similar age and sex 28. Taken together, patients with cardiovascular events have an increased risk of incident diabetes. While insulin resistance is the earliest metabolic abnormality detected in subjects destined to develop diabetes 29, and precipitously increased with hemoglobin A1c levels >5.5%, the CAS patients in our single hospital study had an average glycated hemoglobin level 5.8%, which are considered high-risk for diabetes by the American Diabetes Association 29. In addition, the higher short-term risk of developing diabetes among CAS patients than controls may be attributed to earlier medical attention for CAS. On the other hand, insulin resistance and atherosclerosis could represent independent responses to the disruption of cellular homeostasis 6. Inactivation of the insulin receptor decreases mouse atherosclerosis lesions 6, which become more complex at later time points 6, suggesting that insulin resistance could have differential adaptive effects on CAS and atherosclerotic obstructive coronary artery disease.

The age-associated incidence of CAS-related diabetes was similar to previous studies in patients after stroke 27 and the general population 15. However, age did not affect the risk of CAS-related incident diabetes, suggesting that CAS may be a stronger modulating factor than age for incident diabetes. Sex differences in risk of incident diabetes arise from sociocultural processes, such as stress 15, which has a greater impact on incident diabetes in females than males 15. Therefore, the sex differences in CAS-related diabetes result from the effects other than hormones. Furthermore, when glucose tolerance deteriorates, insulin sensitivity in women is reduced more dramatically than in men 15, contributing to the higher risk of incident diabetes in females than males with CAS, which may partly explain why women show better insulin sensitivity if they are normoglycemic 15. Although dyslipidemia contributes to acquired insulin resistance 30, dyslipidemia did not increase the risk of diabetes in CAS patients, which may be due to the more important role of the reciprocal relationships between impaired insulin-stimulated blood flow and glucose uptake 5 than dyslipidemia in CAS. While COPD with associated insulin resistance has been demonstrated to be a risk factor for incident diabetes 31, CAS had a higher risk of incident diabetes in patients without COPD than with COPD, suggesting that impaired lung function in CAS does not increase the risk of diabetes. Previous studies showed that more than half of non-diabetic stroke patients have pre-diabetes 3 months after stroke 32. However, the risk of incident diabetes was higher in CAS patients without stroke than with stroke when impaired glucose metabolism in the acute stress period should have subsided, suggesting that it is a reflection of unrecognized incident diabetes and not caused by stress of the acute phase of cardiovascular events. Although gout contributes to insulin resistance and increased levels of CRP 33, gout did not increase the risk of CAS-related diabetes. Hence, CRP could be an epiphenomenon in the development of CAS-related diabetes. While hypertension is less closely associated with insulin resistance than are other metabolic abnormalities 34, the antihypertensive treatment improves both insulin sensitivity and endothelial function 13. Therefore, the risk of diabetes was more apparent in CAS patients without hypertension or with non-medicated hypertension than in CAS patients with medicated hypertension. Taken together, the increased risk of incident diabetes associated with CAS was more apparent in females aged <50 years and patients without dyslipidemia, COPD, stroke, gout or medicated hypertension. Prospective studies are needed to examine this hypothesis.

While the incidence of diabetes in the general population reaches the highest rates in the very old females 15, CAS patients aged <50 than ≥50 years have a greater risk of incident diabetes in females but not in males, which seems to be related to sex differences in pre-diabetes 15. Moreover, the essential interaction between estrogen receptor α and caveolin-1 for localization of estrogen receptor to the plasma membrane in endothelial cells 35 may be abrogated in CAS because increased Interleukin-6 levels inhibit endothelial nitric oxide synthase activation by increasing endothelial nitric oxide synthase binding to caveolin-1 in vascular endothelial cells 36. Although the complex interactions between insulin resistance, hyperglycemia and estrogen impairs the endothelial response in females more dramatically than in males 15, as age reduces the benefits of estrogen on insulin-mediated glucose disposal 37 in females, sex differences are reduced. Our data suggest that the sex-specific effects of CAS attenuate the protective effect of female sex, leading to a higher risk of incident diabetes in females than in males.

Consistent with a previous study showing that the rate of onset of diabetes was greater for men than for women 38, the median time to CAS-related incident diabetes was slightly earlier in males rather than females. Moreover, insulin resistance occurs with aging 39. Thus, older rather than younger males with CAS may be less sensitive to insulin, leading to a shorter length of time in developing diabetes. However, age did not reduce the time to incident diabetes in CAS females aged <50 years, suggesting a protective role for premenopausal estrogen against incident diabetes. Although we did not perform any analyses of lifestyle or dietary habits among CAS patients, these factors could be key variables that could affect the length of time before incident diabetes develops between males and females with CAS. Because the early onset of diabetes is associated with a more aggressive diabetic course, a better understanding of the potentially modifiable precursors to cardiometabolic disease, such as CAS, is essential.

Our study has limitations. First, the major limitation is the use of diagnoses in an electronic health record, and thus these diagnoses lack the rigor of a research set of objective definitions. To mitigate the impact of misclassification bias due to coding error, we required that CAS has at least 3 outpatient diagnoses or 1 inpatient diagnosis, and for diabetes, we required ≥4 outpatient visits and the prescription of anti-diabetic drugs. Second, while controlling for confounding factors using multivariate modeling, personal information such as smoking habits and substance use were not available because of the privacy policy governing this database. For consideration of these confounders, socioeconomic indicators such as sex, monthly income and place of residence were adjusted in the regression analyses. However, Taiwan has a particularly high male smoking prevalence and much lower female prevalence among adults with the male-to-female ratio of 11 (46.8% and 4.3%, respectively) 40. Hence, sex could be a proxy variable for smoking. Additionally, the prevalence rates of smoking in patients with diabetes are similar to those of the general population 41, it is essential to address the main risk factor associated with smoking to prevent the onset of diabetes. Third, data on obesity and body mass index were not available in the NHIRD. However, obesity may not affect the temporal relationship between CAS and diabetes because obesity does not contribute to the pathogenesis of CAS 18. Fourth, our study population was a group of potentially health-conscious individuals. Therefore, the timeline for diabetes in our study population could be steeper than that in the general population. Fifth, to maximize specificity, we identified incident diabetes cases based on a diagnosis code and a dispensing code for antidiabetic drugs. Even though we adjusted for 14 demographic, comorbidity and medication variables using propensity score weighting and matching, the study may be subject to residual confounding by unmeasured variables, such as the family history of diabetes, physical activity, menopausal status among women, diet and so on. Sixth, given no ICD-9-CM codes for menopause, future formal clinical studies are required to investigate the time sequence of CAS and menopause as a tool for sex analysis. Seventhly, the biochemistry values like troponin or creatine kinase were not available in NHIRD. However, the panel review system of Taiwan's Bureau of NHI is responsible for auditing laboratory tests, exams, medications, and interventions by comprehensive review of medical records. This auditing system could substantially mitigate the bias carried by overdiagnosis or misdiagnosis. Finally, a limitation of matching is that unexposed individuals not matched to exposed individuals, and possibly some unmatched exposed individuals, are excluded from the analysis, leading to a decrease in the estimated association. However, in our study with many covariates, the propensity score offers a straightforward approach to reduce the dimensionality of the array of confounders.

## Conclusions

CAS, a potentially life-threatening disease, is an important early cardiovascular risk factor for incident diabetes regardless of sex and the length of follow-up. However, CAS patients aged <50 years compared with patients ≥50 years had a greater risk of incident diabetes in females but not in males. Therefore, the diagnosis of CAS is important to detect such sex-specific differences in CAS-related pre-diabetes. Our study suggests that new physiological studies designed to unravel the mechanism of CAS-related incident diabetes are warranted.

## Supplementary Material

Supplementary tables.

## Figures and Tables

**Table 1:** Baseline characteristics of patients with CAS and control subjects without obstructive coronary artery disease

Variable	Data before IPTW^a^		STD	Data after IPTW^b^		STD
	Nondiabetic CAS (n = 12,413)	Nondiabetic Control (n = 94,721)		Nondiabetic CAS	Nondiabetic Control	
Age (years)	56.3 ± 14.3	56.7 ± 15.9	-0.03	57.5 ± 14.7	56.7 ± 15.8	0.05
Male	6,338 (51.1)	48,203 (50.9)	<0.01	50.5	50.9	-0.01
**Urbanization level**						
Low	1,950 (15.7)	10,390 (11.0)	0.14	10.7	11.5	-0.03
Moderate	5,107 (41.1)	26,852 (28.3)	0.27	29.8	30	<0.01
High	3,362 (27.1)	30,746 (32.5)	-0.12	31.1	31.8	-0.02
Very High	1,994 (16.1)	26,733 (28.2)	-0.30	28.5	26.8	0.04
**Monthly income (NTD$)**						
0 - 17,880	3,723 (30.0)	42,610 (45.0)	-0.31	44	43.2	0.02
17,881 - 22,800	4,417 (35.6)	25,499 (26.9)	0.19	27.3	28	-0.02
> 22,800	4,273 (34.4)	26,612 (28.1)	0.14	28.7	28.8	<0.01
**Comorbidity**						
Dyslipidemia	3,012 (24.3)	4,768 (5.0)	0.57	8.5	7.6	0.03
Chronic obstructive pulmonary disease	1,358 (10.9)	3,800 (4.0)	0.27	5.3	4.9	0.02
Stroke	558 (4.5)	2,593 (2.7)	0.09	4	3	0.06
	1,010 (8.1)	3,557 (3.8)	0.19	5.8	4.4	0.06
Hepatitis C virus infection	217 (1.7)	650 (0.7)	0.10	1	0.8	0.02
Depression	325 (2.6)	846 (0.9)	0.13	1.3	1.1	0.02
Psychiatric disorders	41 (0.3)	590 (0.6)	-0.04	0.6	0.6	<0.01
Medicated hypertension	5,125 (41.3)	11,797 (12.5)	0.69	17.6	16.2	0.04
Steroid	284 (2.3)	822 (0.9)	0.11	1.3	1.1	0.02
Follow-up duration, years	5.3 ± 3.4	6.4 ± 3.8	-0.28	6.2 ± 3.6	6.2 ± 3.8	<0.01

**Abbreviations:** IPTW: inverse probability of treatment weighting; CAS: coronary artery spasm; STD: standardized difference. ^a^Data are presented as frequency (percentage) or mean ± standard deviation. ^b^Data are presented as percentage or mean ± standard deviation.

**Table 2:** Incidence of diabetes associated with CAS without obstructive coronary artery disease stratified by sex^a^

Population/follow-up	Nondiabetic CAS			Nondiabetic Control			SHR of CAS^c^ (95% CI)
	No. of Case	ID (95% CI)^b^	ID (95% CI)^c^	No. of Case	ID (95% CI)^b^	ID (95% CI)^c^	
**All**							
1 year	337	29.1 (26.0-32.2)	24.9 (23.9-25.8)	1,445	15.7 (14.9-16.5)	17.2 (16.4-18.0)	1.45 (1.37-1.54)
3 year	733	24.1 (22.3-25.8)	21.4 (20.9-22.0)	3,380	13.6 (13.2-14.1)	14.6 (14.1-15.0)	1.39 (1.35-1.43)
5 year	1,026	23.4 (21.9-24.8)	21.2 (20.7-21.6)	4,898	13.3 (12.9-13.6)	14.1 (13.7-14.4)	1.53 (1.48-1.58)
Overall	1,434	23.5 (22.3-24.7)	22.2 (21.8-22.6)	7,593	13.3 (13.0-13.6)	13.9 (13.6-14.2)	1.61 (1.57-1.65)
**Male**							
1 year	176	29.8 (25.4-34.2)	25.7 (24.3-27.1)	761	16.4 (15.2-17.5)	17.6 (16.5-18.8)	1.47 (1.35-1.60)
3 year	375	24.2 (21.8-26.7)	21.6 (20.8-22.3)	1,793	14.4 (13.7-15.0)	15.3 (14.6-15.9)	1.31 (1.25-1.37)
5 year	506	22.7 (20.7-24.6)	20.3 (19.7-20.9)	2,532	13.7 (13.1-14.2)	14.4 (13.9-14.9)	1.42 (1.35-1.49)
Overall	723	23.2 (21.5-24.9)	21.6 (21.1-22.2)	3,990	14.0 (13.5-14.4)	14.6 (14.2-15.0)	1.50 (1.44-1.56)
**Female**							
1 year	161	28.4 (24.0-32.8)	23.5 (22.2-24.9)	684	15.0 (13.9-16.2)	16.6 (15.5-17.7)	1.42 (1.30-1.55)
3 year	358	23.9 (21.4-26.4)	20.8 (20.0-21.6)	1,587	12.9 (12.3-13.5)	13.9 (13.2-14.5)	1.49 (1.43-1.55)
5 year	520	24.1 (22.0-26.1)	21.7 (21.1-22.4)	2,366	12.9 (12.4-13.4)	13.6 (13.1-14.1)	1.62 (1.54-1.70)
Overall	711	23.8 (22.1-25.6)	22.6 (22.0-23.1)	3,603	12.6 (12.2-13.0)	13.2 (12.8-13.6)	1.73 (1.66-1.80)

**Abbreviations:** CAS: coronary artery spasm; ID: incidence density; CI: confidence interval; SHR: subdistribution hazard ratio. ^a^The inverse probability of treatment weight was calculated separately in each sex. ^b^Incidence density: number of events per 1000 person-years. ^c^Adjusted for inverse probability of treatment weighting by propensity score.

## Abbreviations

CAS: coronary artery spasm
CI: confidence interval
COPD: chronic obstructive pulmonary disease
CRP: C-reactive protein
ICD-9-CM: International Classification of Diseases, Ninth Revision, Clinical Modification
IPTW: inverse probability of treatment weighting
NHI: National Health Institute
NHIRD: National Health Insurance Research Database
SHR: subdistribution hazard ratio
