# Supplementary material for: Association between Coronary Artery Spasm and the risk of incident Diabetes: A Nationwide population-based Cohort Study
Source: Int J Med Sci. 2021 May 3;18(12):2630–40. doi: 10.7150/ijms.57987 (PMC8176166; doi:10.7150/ijms.57987)
Supplement: Supplementary file 1 — Supplementary tables. [file ijmsv18p2630s1.pdf]

Supplementary Tables

Table S1. Baseline characteristics of CAS and control subjects without obstructive CAD in a single hospital.

Variable	Non-diabetic Non-CAS (n = 112)	Non-diabetic CAS (n = 140)	*P* value
Age (years)	52.8 ± 11.8	56.9 ± 11.8	0.007
Male	53 (47.3)	90 (64.3)	0.007
Body mass index (kg/m^2)	26.6 ± 5.3	26.1 ± 3.7	0.428
Blood pressure (mmHg)			
Systolic	130.1 ± 20.3	122.6 ± 18.9	0.004
Diastolic	77.3 ± 12.5	74.4 ± 10.8	0.060
Heart rate (beats/min)	73.5 ± 15.1	66.0 ± 10.3	<0.001
Current smoker	24 (21.4)	47 (33.6)	0.033
Hypertension	41 (36.6)	50 (35.7)	0.883
Left ventricular ejection fraction, %	66.0 ± 8.5	66.1 ± 7.3	0.910
Total cholesterol (mg/dL)	184 ± 40	186 ± 41	0.797
Triglyceride (mg/dL)	155 ± 137	148 ± 118	0.659
LDL cholesterol (mg/dL)	91.0 ± 52.9	97.6 ± 51.1	0.326
HDL cholesterol (mg/dL)	36.7 ± 21.2	38.1 ± 19.2	0.598
Peripheral leukocytes (x cells/mm^3)	6874 ± 1836	7380 ± 2206	0.053
Monocytes (x cells/mm^3)	506 ± 190	541 ± 195	0.155
Eosinophils (x cells/mm^3)	146 ± 100	189 ± 180	0.024
Hemoglobin (g/dL)	13.5 ± 1.8	13.7 ± 1.6	0.212
Hematocrit (%)	39.7 ± 4.9	40.5 ± 4.4	0.170
Platelet (×10^3 cells/mm^3)	230 ± 59	225 ± 61	0.499
Fasting glucose (mg/dL)	98 ± 11	102 ± 16	0.015
HbA1c (%)	5.6 ± 0.7	5.8 ± 0.7	0.041

Variable	Non-diabetic Non-CAS (n = 112)		Non-diabetic CAS (n = 140)		*P* value	
hs-CRP (mg/L[a])	0.79 (0.41, 1.63)		0.94 (0.44, 2.98)		0.031	
Provoked coronary artery						
Left anterior descending artery			59 (42.5)			
Left circumflex artery			33 (23.8)			
Right coronary artery			105 (75.5)			
Number of spastic arteries						
One-vessel			98 (62.8)			
Two-vessels			22 (14.1)			
Three-vessels			18 (11.5)			
Medications	A	D	A	D	A	D
β–Blockers	38 (34.5)	18 (16.4)	67 (47.9)	23 (16.4)	0.034	0.989
Calcium channel blockers	41 (36.9)	34 (30.6)	50 (35.7)	110 (78.6)	0.841	<0.001
Angiotensin receptor blocker	41 (36.9)	41 (36.6)	50 (35.7)	46 (32.9)	0.841	0.534
Nitrates	13 (11.6)	10 (8.9)	36 (25.7)	10 (7.1)	0.005	0.602
Statins	6 (5.4)	5 (4.5)	2 (1.4)	2 (1.4)	0.077	0.145
Aspirin	82 (73.2)	36 (32.1)	103 (73.6)	109 (77.9)	0.949	<0.001

Abbreviations: A: before angiography; CAD: coronary artery disease; CAS: coronary artery spasm; D: at discharge; HbA1c: glycated hemoglobin; LDL: low density lipoprotein; HDL: low density lipoprotein; hs-CRP: high-sensitivity C-reactive protein.

[a]Log-transformed values were used in analyses.

Table S2. Sensitivity analysis for accounting for potential detection bias by adding a one-year lag period.[a]

	Nondiabetic CAS			Nondiabetic Control			
Follow-up	No. of Case	ID (95% CI)[b]	ID (95% CI)[c]	No. of Case	ID (95% CI)[b]	ID (95% CI)[c]	SHR of CAS[c] (95% CI)
3 year	396	13.0 (11.7–14.3)	12.3 (11.8–12.7)	1,935	7.8 (7.5–8.2)	8.2 (7.8–8.5)	1.32 (1.27–1.38)
5 year	689	15.7 (14.5–16.9)	15.0 (14.6–15.4)	3,453	9.4 (9.0–9.7)	9.7 (9.4–10.0)	1.56 (1.50–1.62)
Overall	1,097	18.0 (16.9–19.1)	18.0 (17.7–18.4)	6,148	10.8 (10.5–11.0)	11.1 (10.8–11.3)	1.65 (1.60–1.70)

Abbreviations: CAS: coronary artery spasm; ID: incidence density; CI: confidence interval; SHR: subdistribution hazard ratio.

[a]Patients who were diagnosed as diabetes within 1 year after the index date were censored.

[b]Incidence density: number of events per 1000 person-years.

[c]Adjusted for inverse probability of treatment weighting by propensity score.

Table S3. Age-specific incidence of diabetes associated with nondiabetic CAS in the whole cohort stratified by sex.

	Total	Male	Female
Age group, year	ID (95% CI)[a]	ID (95% CI)	ID (95% CI)
20-29	4.6 (1.9–7.4)	4.5 (0.6–8.4)	4.8 (1.0–8.6)
30-39	9.6 (7.1–12.2)	10.3 (6.8–13.7)	8.8 (5.1–12.5)
40-49	18.0 (15.7–20.3)	19.4 (16.2–22.6)	16.4 (13.2–19.5)
50-59	28.0 (25.4–30.6)	29.2 (25.5–33.0)	26.7 (23.1–30.3)
60-69	28.5 (25.6–31.4)	27.2 (23.2–31.3)	29.7 (25.5–33.9)
70-79	31.0 (27.2–34.7)	27.2 (22.1–32.3)	34.4 (28.9–40.0)
80-89	23.9 (17.2–30.6)	23.1 (13.9–32.3)	24.7 (15.0–34.3)
≥90	49.0 (6.0–91.9)	93.3 (-36.0–222.7)	37.2 (-4.9–79.3)

Abbreviations: ID: incidence density.

[a]Incidence density: number of events per 1000 person-years.

Table S4. Baseline characteristics of patients with CAS and control subjects without obstructive coronary artery disease before and after propensity score matching.

	Data before matching			Data after matching		
Variable	Nondiabetic CAS (n = 12,413)	Nondiabetic Control (n = 94,721)	STD	Nondiabetic CAS (n = 12,074)	Nondiabetic Control (n = 12,074)	STD
Age (years)	56.3 ± 14.3	56.7 ± 15.9	-0.03	56.3 ± 14.3	57.0 ± 15.9	-0.05
Male	6,338 (51.1)	48,203 (50.9)	<0.01	6,142 (50.9)	6,180 (51.2)	-0.01
Urbanization level						
Low	1,950 (15.7)	10,390 (11.0)	0.14	1,859 (15.4)	1,933 (16.0)	-0.02
Moderate	5,107 (41.1)	26,852 (28.3)	0.27	4,922 (40.8)	4,991 (41.3)	-0.01
High	3,362 (27.1)	30,746 (32.5)	-0.12	3,306 (27.4)	3,159 (26.2)	0.03
Very High	1,994 (16.1)	26,733 (28.2)	-0.30	1,987 (16.5)	1,991 (16.5)	<0.01
Monthly income (NTD$)						
0 - 17,880	3,723 (30.0)	42,610 (45.0)	-0.31	3,680 (30.5)	4,022 (33.3)	-0.06
17,881 – 22,800	4,417 (35.6)	25,499 (26.9)	0.19	4,261 (35.3)	4,267 (35.3)	<0.01
> 22,800	4,273 (34.4)	26,612 (28.1)	0.14	4,133 (34.2)	3,785 (31.3)	0.06
Comorbidity						
Dyslipidemia	3,012 (24.3)	4,768 (5.0)	0.57	2,691 (22.3)	2,624 (21.7)	0.01
Chronic obstructive pulmonary disease	1,358 (10.9)	3,800 (4.0)	0.27	1,244 (10.3)	1,252 (10.4)	<0.01
Stroke	558 (4.5)	2,593 (2.7)	0.09	535 (4.4)	599 (5.0)	-0.03
Gout	1,010 (8.1)	3,557 (3.8)	0.19	927 (7.7)	1,028 (8.5)	-0.03
Hepatitis C virus infection	217 (1.7)	650 (0.7)	0.10	200 (1.7)	204 (1.7)	<0.01
Depression	325 (2.6)	846 (0.9)	0.13	285 (2.4)	280 (2.3)	<0.01
Psychiatric disorders	41 (0.3)	590 (0.6)	-0.04	41 (0.3)	46 (0.4)	-0.01

	Data before matching			Data after matching		
Variable	Nondiabetic CAS (n = 12,413)	Nondiabetic Control (n = 94,721)	STD	Nondiabetic CAS (n = 12,074)	Nondiabetic Control (n = 12,074)	STD
Medicated hypertension	5,125 (41.3)	11,797 (12.5)	0.69	4,793 (39.7)	5,051 (41.8)	-0.04
Steroid	284 (2.3)	822 (0.9)	0.11	258 (2.1)	273 (2.3)	-0.01
Follow-up duration, years	5.3 ± 3.4	6.4 ± 3.8	-0.28	5.4 ± 3.4	5.2 ± 3.4	0.06

Abbreviations: CAS: coronary artery spasm; STD: standardized difference.

Data are presented as frequency (percentage) or mean ± standard deviation.

Table S5. Incidence of diabetes associated with CAS without obstructive coronary artery disease stratified by sex in the propensity score matched cohort[a].

	Nondiabetic CAS		Nondiabetic Control		
Population	No. of Case (%)	ID (95% CI)[ab]	No. of Case (%)	ID (95% CI)[ab]	SHR of CAS (95% CI)
Overall	1,391 (11.5)	23.3 (22.0–24.5)	1,121 (9.3)	19.1 (18.0–20.2)	1.25 (1.15–1.35)
Male	689 (11.3)	22.8 (21.1–24.5)	554 (9.1)	18.7 (17.1–20.2)	1.25 (1.12–1.40)
Female	697 (11.7)	23.6 (21.8–25.3)	529 (8.9)	18.2 (16.6–19.7)	1.32 (1.18–1.48)

Abbreviations: CAS: coronary artery spasm; ID: incidence density; CI: confidence interval; SHR: subdistribution hazard ratio.

[a]The propensity score was calculated separately in each sex.

[b]Incidence density: number of events per 1000 person-years.
